# Thyroid Autoimmunity and Autoimmunity in Chronic Spontaneous Urticaria Linked to Disease Severity, Therapeutic Response, and Time to Remission in Patients with Chronic Spontaneous Urticaria

**DOI:** 10.1155/2018/9856843

**Published:** 2018-11-01

**Authors:** Kumutnart Chanprapaph, Wimolsiri Iamsumang, Penpun Wattanakrai, Vasanop Vachiramon

**Affiliations:** Division of Dermatology, Department of Internal Medicine, Ramathibodi Hospital, Mahidol University, Bangkok, Thailand

## Abstract

**Background:**

Chronic spontaneous urticaria (CSU) is autoimmune in nature and associated with thyroid autoimmunity (TA), but evidence on autoimmunity in relation to CSU progression and prognosis is limited. We evaluated whether TA and autoimmunity in CSU are correlated with disease severity, therapeutic response, and time to remission and establish an association between CSU characteristics linked to thyroid autoantibody.

**Methods:**

Medical records of patients diagnosed with urticaria attending outpatient dermatology clinic at a university-based hospital from 2013 to 2017 were retrospectively reviewed. Data on the clinical characteristics, laboratory investigations particularly thyroid antibody titers, autologous serum skin test (ASST) and autologous plasma skin test (APST) results and their link to disease severity, treatments, and time to remission of CSU patients were analyzed.

**Results:**

Of 1,096 patients with urticaria, 60.2% had CSU. Three-hundred patients fulfilled the inclusion criteria for CSU with complete thyroid antibody testing. Positive TA was significantly associated with female gender and age > 35 years (p = 0.008). Antithyroid peroxidase (anti-TPO)-positive patients suffered from CSU longer than 12 and 18 months compared to anti-TPO-negative patients (100.0% vs. 82.6%, p = 0.042, and 100.0% vs. 75.9% p = 0.020, respectively). The presence of urticarial attacks > 4 days/week was significantly seen in ASST and APST-positive patients compared to those without (84.6% vs. 61.3%, p = 0.011, and 85.3% vs. 61.8%, p = 0.006, respectively). Positive APST patients were more difficult to treat than those with negative results (61.2% vs. 37.8%, p = 0.017).

**Conclusions:**

Antithyroid peroxidase is a predictor of time to remission, while autologous skin testing is linked to disease severity (ASST and APST) and therapeutic response (APST) in CSU patients.

## 1. Introduction

Thyroid autoimmunity (TA) is characterized by the production of thyroid autoantibodies and lymphocytic infiltration into the thyroid glands. It is the most common organ-specific disorder affecting approximately 5% of the general population [1, 2]. Positive thyroid autoantibody is essential for the diagnosis of TA. As the exact pathogenesis is unclear, hereditary and environmental factors appear to be fundamental processes of TA [1].

Chronic spontaneous urticaria (CSU) is defined as the presences of recurrent wheals and flare for a duration of 6 weeks independent of external stimuli [3]. CSU is a common cutaneous disorder with an estimated prevalence of 8-10% of the general population [4]. CSU has major undesirable effects and significantly impacts the quality of life, mainly due to the high disease activity, sleep deprivation, and psychiatric comorbidity. Therefore, determining factors linking to the severe and resistant cases of CSU is important, as it allows physicians to be more aggressive on their management plans. Majority of cases with CSU have unknown etiology with approximately 30-40% have autoimmune pathogenesis [5]. Assessing for autoreactivity in-vivo via autologous serum skin test (ASST) and autologous plasma skin test (APST) and in-vitro through basophil histamine release and basophil activation test (BAT) are widely applied. While there is evidence to show that BAT with or without the combination of ASST can identify patients with more severe CSU [6, 7], there is limited data on whether these results can predict therapeutic response and time to remission in CSU. Coexistence of CSU with major autoimmune diseases has been well documented, particularly autoimmune thyroid diseases (AITD) [8]. The prevalence of positive thyroid autoantibodies in patients with urticaria is significantly higher than nonurticaria controls [1]. Likewise, a recent population-based study has shown that patients with AITD has higher rate of CSU [9].While the association between TA and CSU is well known and is one of the clinical association that contribute to autoimmune hypothesis [6], the relationship between antithyroid antibody and the progression and prognosis of CSU is largely unknown. The objective of this study is to determine the association between TA and autoimmunity of CSU in relation to CSU disease severity, therapeutic response, and time to remission and establish an association between CSU characteristics linked to thyroid autoantibody.

## 2. Material and Methods

### 2.1. Study Design

A retrospective study was conducted in a university-based hospital (Ramathibodi Hospital, Mahidol University, Bangkok, Thailand). The medical records of all patients diagnosed with urticaria visiting outpatient dermatologic clinic from January 2013 to May 2017 were retrieved and analyzed. The study was approved from the Mahidol University Institution Review Board (IRB) for human subject research (protocol number 076036). Informed consent was exempted by the board due to the retrospective nature of the study.

### 2.2. Subjects

Individuals ≥ 15 years of age who met the diagnostic criteria of CSU, having recurrent wheals and flare of less than 24 hours occurring at least 2 times per week for 6 weeks without identifiable causes, were enrolled in the study. Patients with inducible urticaria (i.e., physical, pressure, cholinergic, cold, drug-induced, and acute urticaria) were excluded. Cases suspected for or had skin biopsy-proven urticarial vasculitis were also excluded from the study. Patients lacking information on both autoimmune thyroid antibodies, including anti-TPO and anti-Tg, were excluded.

### 2.3. Protocol

Medical record forms were collected for clinical and laboratory information. Data were entered into a database program (Microsoft Excel 2013; Microsoft Corp, Redmond, Washington). Clinical parameters involving patients' gender, age, duration of disease, previous history of AITDs, atopy, systemic symptoms (i.e. angioedema, anaphylaxis), dermographism together with disease severity, therapeutic response, and time to remission were collected. Patients were evaluated for disease severity focusing on duration and frequency of daily attacks, wheal size and number, severity of itch, impairment of work, and disturbance of sleep. Therapeutic response was determined by treatment regimens used, detailed types and dosages of antihistamines, and other medications (H2-receptor antagonist, antileukotrienes, cyclosporin A, omalizumab) were reviewed. Individuals unresponsive to the standard doses of the second generation H1-antihistamines were categorized as difficult-to-treat cases. The length of disease duration after treatment was recorded and remission rate at 12 and 18 months were calculated. The duration from the onset of CSU with the presence of thyroid autoantibody to the development of AITD was evaluated. A review of laboratory tests related to urticaria were conducted (i.e. Complete blood count, erythrocyte sedimentation rate (ESR), antinuclear antibody (ANA), anti-TPO, anti-Tg, urine analysis (UA), and stool exam). ANA was performed by indirect immunofluorescent technique (EUROPattern®, Euroimmun AG, Luebeck, Germany), a positive test was considered by titer >1:80. Anti-TPO and anti-TG were performed by electro-chemiluminescence immunoassay (Elecsys®, Roche Diagnostics GmbH, Mannheim, Germany). Results were positive if anti-TPO titer > 34 IU/mL or anti-TG > 115 IU/mL. Patients were categorized as having TA if at least one anti-thyroid antibody was positive. Those with TA were further evaluated for thyroid function test (TFT). TFT (Abbott Diagnostics, Lake Forest, IL, USA), included thyroid stimulating hormone (TSH, reference range, 0.35-4.94 uIU/mL), free triiodothyronine (FT3, reference range, 1.71-3.71 pg/mL), and free thyroxine (FT4, reference range, 0.7-1.48 ng/dL). AITDs were diagnosed by endocrinologists. Hashimoto's thyroiditis was diagnosed based on the demonstration of circulating thyroid antibodies and diffuse thyroid enlargement or reduced echogenicity on thyroid ultrasonography. The diagnosis of Graves' disease relies on persistent hyperthyroidism with positive thyroid antibody and/or increase vascularization on thyroid sonogram. The diagnosis of subclinical thyroid diseases was made when serum free T4 and free T3 levels remain within their respective reference ranges with the presence of abnormal TSH levels.

Regarding skin testing, ASST and APST were utilized as an in-vivo test to diagnose chronic autoimmune urticaria (CAU). Antihistamines were withheld 7 days prior to testing. Ten milliliters of venous blood were drawn to prepare the autologous serum and plasma. To perform the skin testing, 0.05 ml of the autologous serum and plasma were injected intradermally into the volar side of each forearm. A negative control was done by using the same technique with 0.05 ml of normal saline (NSS). Skin test reading was performed 30 minutes after the injections. ASST and APST were considered positive with induction of wheal diameter exceeded that of NSS by 1.5 mm.

### 2.4. Statistical Analysis

Statistical analyses were conducted by STATA statistical software version 13 (Stata Corp LP, College station, TX, USA). To test for associations, the statistical methods such as Pearson's Chi-squared test and Fisher's exact test were used for categorical variables, while Student's t-test and Wilcoxon rank-sum test were operated for continuous variables with normal and non-normal distribution, respectively. Statistical significance was considered when p-value < 0.05.

## 3. Results

Medical records of 1,096 patients diagnosed with urticaria were retrospectively reviewed, 463 patients were excluded due to the diagnosis of acute urticaria, urticarial vasculitis, inducible urticaria. Six-hundred sixty patients satisfied the diagnostic criteria for CSU (60.2%), while 360 patients were excluded due to lack of one or both thyroid antibody results, giving a total of 300 study participants fulfilling the study inclusion criteria.

### 3.1. Patient Demographics

The majority of patients were female (84.7%). Female gender was associated with TA, elevated anti-TPO level, and elevated anti-Tg level with statistical significance (*p* = 0.003,* p* = 0.010, and* p* = 0.024, respectively). The mean age of CSU onset was 41.3 ± 14.9 years. Positive TA was significantly associated with CSU onset after 35 years of age compared to earlier age onset (75% vs 57.6%,* p *= 0.008). Fifty-four patients suffered from CSU with the presence of TA at latter onset. Of which 22.2% (12 patients) had AITDs consisting of Graves' disease 11.1% (6 patients), Hashimoto's thyroid disease 5.6 % (3 patients), subclinical hypothyroidism 3.7% (2 patients), and subclinical hyperthyroidism 1.9% (1 patient). The association between the onset of CSU and positive anti-Tg followed a similar direction (78.2% vs. 58.1%, respectively,* p* = 0.006). The same trend was shown for positive anti-TPO but without statistical significance (69.8% vs. 60.1%,* p* = 0.187). The disease duration ranged from 1.5-360 months (median 4 months). For atopic diathesis, allergic rhinitis was the most frequently reported (54.9%). The occurrences of patients with history of nonsteroidal anti-inflammatory drug hypersensitivity, atopy, and dermographism, although not statistically significant, were higher in patients with TA. The parameters for CSU severity including frequency of attacks, wheal size, number of wheals, severity of itch, impairment of work, and disturbance of sleep were reported in percentage in Table 1.

In contrast to TA positivity, higher percent of CAU patients developed CSU before or at the age of 35 years compared to non-CAU (62.4% vs. 37.5%,* p* = 0.027). With statistical difference, approximately 64% of ASST-positive patients had history of atopy in comparison to 36.4% of ASST-negative patients (*p* = 0.032). The presence of angioedema was more commonly noted in patients with CAU, ASST and APST positivity compared to those without CAU, or with negative ASST and APST (*p* = 0.058,* p* = 0.183, and* p* = 0.085, respectively), shown in Table 2.

### 3.2. Laboratory Analyses

**Figure 1:**
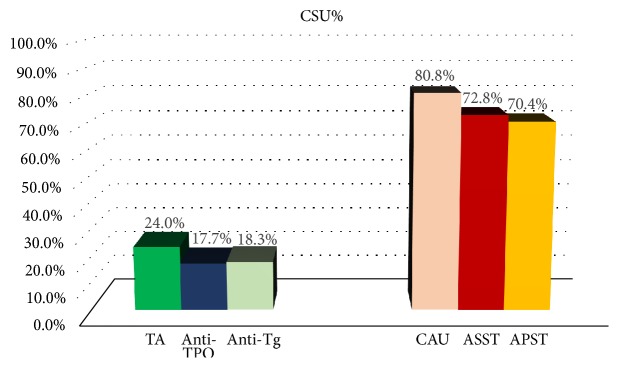
Percentages of chronic spontaneous urticaria patients who had thyroid autoimmunity (TA), positive anti-TPO (antithyroid peroxidase), positive anti-Tg (anti-thyroglobulin), chronic autoimmune urticaria (CAU), positive autologous serum skin test (ASST), and positive autologous plasma skin test (APST).

**Figure 2:**
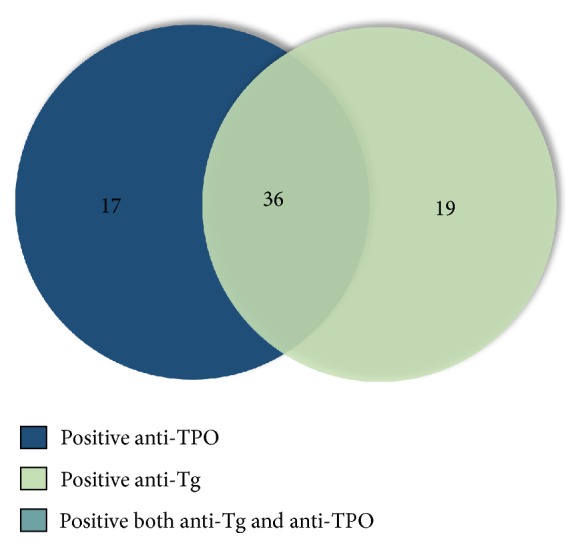
Number of CSU patients with positive antithyroid peroxidase (anti-TPO) and antithyroglobulin (anti-Tg).

Details of related laboratory findings are shown in Table 1. Anti-TPO and anti-Tg revealed positive for 17.7% and 18.3%, respectively. Twenty-four percent had either anti-TPO or anti-Tg positivity while both tests were positive in 36 patients. (Table 2, Figures 1 and 2). The frequency of high-titer-ANA-positive patients was significantly correlated with higher frequency of TA, positive anti-TPO, and positive anti-Tg (*p* = 0.015,* p* = 0.002, and* p* = 0.046, respectively). The frequency of patients showing elevated ESR was significantly higher in patients with TA and anti-TPO positivity than those without (*p* = 0.035,* p* = 0.024, respectively).

**Figure 3:**
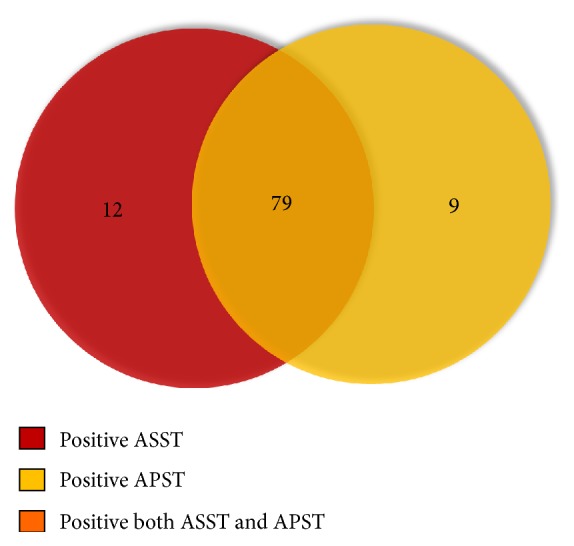
Number of CSU patients with positive autologous serum skin test (ASST) and autologous plasma skin test (APST).

Autologous skin testing was carried out in 125 CSU patients, 101 (80.8%) including 88 females (87.1%) and 13 males (12.9%) has positive results (Table 2, Figure 1). Both tests were positive in 79 patients (Figure 3). ASST and APST were positive in 91(72.8%) and 88 (70.4%) patients, respectively (Figures 1 and 3).The median time to perform the skin tests was 13 months (range 1.5-365 months) after CSU onset. There was no statistically significant association between TA and CAU (*p* = 0.316) (Tables 1 and 2).

### 3.3. Thyroid Autoimmunity in Patients with CSU

Of 300 patients, high anti-TPO and anti-Tg were detected in 53 and 55 patients, respectively, and both thyroid autoantibodies were elevated in 12% (Figure 2). Seventy-two (24%) patients with positivity to any thyroid autoantibodie(s) were diagnosed with TA and were subsequently evaluated for TFT. At the median time of 5 months from the CSU onset, AITDs were established in 10 patients (patient number 8, 10-12, and 14-18). By periodically repeating TFT, two more patients (patient number 9 and 13) were diagnosed with AITDs at the median time of 21 months from CSU onset. AITDs which corresponded to 15.3% (18/72; excluding patient number 3 due to negative thyroid autoantibodies) of patients showed positivity to either anti-TPO or anti-Tg. Seven patients suffered from AITDs prior to the onset of CSU (patient number 1-7). Information of CSU patients with AITDs is demonstrated in Table 3. All patients with AITDs were female. Graves' disease was the most common, followed by Hashimoto's disease. Other diagnoses included subclinical hypothyroidism, subclinical hyperthyroidism, and primary hypothyroidism.

### 3.4. Thyroid Autoantibodies and Autoimmunity in CSU as a Predictor of CSU Prognosis

#### 3.4.1. Disease Severity

Generally, previously mentioned clinical parameters (see Methods) for CSU severity were not statistically different between patients with TA and the presence of anti-TPO and anti-Tg compared to those without.

As shown in Table 2, urticarial attacks > 4 days/week was more frequently reported in ASST- and APST-positive patients compared to ASST- and APST-negative patients (84.6% vs. 61.3%,* p* = 0.011, and 85.3% vs. 61.8%,* p* = 0.006). CAU patients had greater percentage of attacks by CSU > 4 days/week than non-CAU patients but the statistical significance was not reached (*p* = 0.086). Likewise, 73.9% of patients with CAU experienced the number of wheals > 7 lesions/day compared to 42.9% of those without (*p *= 0.051). In addition, the mean wheal diameter induced by autologous plasma was significantly associated with number of wheals > 7 lesions/day (9.4 mm vs. 7.9 mm,* p* = 0.037). Other clinical and laboratory indicators for severity including wheal size, severity of itch and disturbance of sleep were not significantly correlated with either the presence of CAU, positive ASST, or positive APST.

#### 3.4.2. Therapeutic Response

Second-generation H1-antihistamines exceeding standard dosages either given singly or in combination with other H1-antihistamine(s) were administered in over half of the patients (57.0%) while the rest were controlled with standard-dose-H1-antihistamine (Table 1). Cyclosporin A, omalizumab, monteleukast, and H2-receptor antagonist were prescribed in 2, 1, 3, and 17 patients, respectively. The presence of TA, anti-TPO, or anti-Tg did not significantly influence therapeutic response (*p* = 0.667,* p* = 0.296, and* p* = 0.819, respectively).

In terms of CAU, patients with positive APST had higher frequency of commencing second generation H1-antihistamine(s) exceeding standard dosages compared to negative APST (61.2% vs. 37.8%,* p* = 0.017). The association of CAU and difficult-to-treat cases showed similar pattern but did not reach statistical significance (*p* = 0.069), however, ASST did not demonstrate differences in therapeutic regimens (*p* = 0.873) (Table 2).

#### 3.4.3. Time to Remission

As shown in Table 1, 85.5% and 80.2% suffered from persistent CSU of more than 12 and 18 months, respectively. Patients with TA had active disease longer than 12 and 18 months after therapy compared to those without (93.3% vs. 83.2% and 92.0% and 76.3%, respectively) though the statistical difference was not reached (*p* = 0.240 and* p* = 0.088, respectively). A similar pattern was observed in anti-Tg-positive and anti-Tg-negative group. However, anti-TPO-positive patients were significantly more prevalent in attaining persistent disease longer than 12 and 18 months (100% vs. 82.6%, p = 0.042 and 100% vs. 75.9%,* p* = 0.020, respectively).

The percentages of patients with active CSU at 12 or 18 months, in CAU or non-CAU group, in ASST-positive or ASST-negative group, or in APST-positive or APST-negative group were not significantly different (Table 2).

## 4. Discussion

The clinical spectrum and outcome of patients with urticaria is variable, hence, multiple factors may be involved in the clinical and prognostic polymorphism. Early predictions on CSU as to whom would be expected to have severe, difficult-to-treat and/or long-lasting disease is desirable for proper patient education and appropriate management plans, therefore, our study is among the few to evaluate these factors in association to TA and autoimmunity in CSU.

Of 1,096 patients, 60.2% fulfilled the criteria for the diagnosis of CSU. The prevalence corresponds to the previous reports of 56-93%. The frequency of TA was 24.0% (17.7% for anti-TPO and 18.3% for anti-Tg) and 6.3% had AITDs (Figure 1). Literature reports on the prevalence of TA ranged from 4.3-57%, among this 5-10% have clinically apparent thyroid disease [10, 11]. In a national survey of normal Thai patients, anti-TPO and anti-Tg were positive in 8.9% and 12.3%, respectively [11]. Our study confirms higher prevalence of TA in the CSU population. We also highlight that AITDs could manifest as early as 9 years prior to or up to 5 years following CSU onset (Table 3). More importantly, many patients were diagnosed by subsequent TFT evaluation. We emphasize that periodic evaluation of TFT is crucial in CSU patients showing positive thyroid autoantibodies. The definite mechanism behind the association between TA and urticaria remains to be determined. However, postulated hypothesis for autoimmune CSU involves two mechanisms: type I and II autoimmunity [12]. In type I autoimmune CSU, IgE autoantibodies bind to high affinity mast cell receptor. Anti-TPO is one of the most common CSU-associated autoallergen identified [13]. In type II autoimmune CSU, particularly autoimmunity type IIb, IgG autoantibody is responsible for mast cell degranulation via activation of high-affinity IgE receptor. The definitive diagnosis of this type relies on positive autologous skin test and/or BAT and histamine release test [13]. Evidence have shown that there is a strong link between elevated levels of IgG antithyroid antibodies and CSU [14]. Therefore, autoimmune CSU in our subpopulation could possibly be classified as autoimmunity type IIb. Genetic and environmental factors could also be attributed. Another possible pathogenesis is that antithyroid drugs, i.e., methimazole, carbimazole, or propylthiouracil, may cause itching and urticaria as seen in several patients in the present study commencing these medication prior to the onset of CSU (Table 3) [15]. Moreover, exposure to specific circulating antigen particularly as a result of autoimmune thyroid damage, anti-TPO IgE is produced and may potentially induce urticarial symptoms, mast cell sensitization and degranulation [16]. Nevertheless, to date, there is insufficient evidence to prove that thyroid autoantibodies are pathogenic for CSU and studies have failed to demonstrate cross-reactivity between antithyroid antibody and other autoantibodies in CSU. In addition, antithyroid antibodies alone are not capable of inducing mast cell activation [17].

In agreement with other reports, TA is more prevalent in females (female: male 5.5:1) [18–20]. The proposed mechanisms for female preponderance may involve the underlying state of inflammation driven by adipokines, especially leptin, TNF-*α*, and IL-6, and several receptors including Toll-like receptors on thyrocytes. Because leptin levels are higher in females, the function of thyrocytes in innate immunity fails to act properly against triggers such as viruses, bacteria, and stress. This then contributes to the initiation step to break tolerance to thyroid self-antigens [19]. The mean age onset of our patients was 41.3 ± 14.9 years. This was similar to that demonstrated in the literature [18, 20]. To the best of our knowledge, we are the first to report the statistically significant association between the presence of TA and the age of CSU onset older than 35 years. Regarding ANA testing, the percentage of patients showing positive ANA titer >1:320 was slightly lower than that of a recent report (17.5%) [20]. This difference can be partially justified by different groups of population and ethnicity. Of note, our study highlights the significant correlation between TA and positive high-titer ANA among CSU patients. This supports earlier studies in CSU showing the association between ANA and TA [21, 22]. It is believed that the presence of non-organ-specific autoantibodies such as ANA, may demonstrate a polyclonally accelerated production of autoantibodies by immune cells and also thyrocytes [22]. However, the presence of ANA titer ≥ 1:320 can be found in 1.4% of the healthy population [23]. Moreover, of 32 patients with positive high-titer ANA, only 2 were diagnosed with lupus erythematosus. Therefore, the presence of positive ANA may not necessarily be pathologic and the clinical significance of ANA positivity remains to be determined.

Our findings suggested that early age onset of before 35 years may predict autoimmune basis of CSU. This is compatible with the earlier study reporting that patients with CAU were relatively younger than non-CAU patients [24]. Sharing IgE-mediated mechanism, CSU is believed to be associated with allergic dysentery. We found a strong association between atopy and ASST positivity. Reports in the literature are mostly but not always consistent with this [24, 25]. Regarding ANA positivity, although strong association was lacking, it was more prevalent in CAU patients. Larger number of participants may yield more apparent results.

Our study demonstrated a relatively higher frequency of CAU patients (80.8%) and also ASST (72.8%) and APST positivity (70.4%) compared to previous literature [25–27]. However, similar to our findings, reports of considerably high positivity for ASST (66%) and APST (86%) have been demonstrated [28, 29]. An explanation for these discrepancies could be from differences in the study population, patient selection, and criteria adopted to score the test [30]. Unlike some reports, we did not find an association between positive autologous skin testing and the presence of antithyroid antibodies [18, 31]. However, our finding is consistent with those of Kocatürk, Yadav, and Alpay et al. [32–34]. Therefore, the association between CAU and TA remains controversial and requires further validation. The presence of TA or positive anti-TPO could serve as a predictor for elevated ESR but not for other clinical parameters. We believe that it is appropriate to monitor ESR in patients with CSU which represents a chronic inflammatory condition. Our study showed that at the end of 12 months, symptoms of CSU persisted in 85.5% of participants and 80.2% existed after 18 months. These figures are relatively high compared to previous reports [27, 35, 36]. This could be due to the high referral rate and more severe and persistent CSU patients sent to our specialized outpatient clinic. Of the thyroid antibodies, anti-TPO alone played a significant role in predicting persistent disease of CSU. An explanation to longer CSU duration related to thyroid antibody remains unclear. A possible mechanism is that long-lasting CSU may result from prolonged T-cell stimulation followed by extended polyclonal activation and the production of various inflammatory mediators. This could possibly induce the production of other autoantibodies such as anti-TPO. Toubi et al. also found that thyroid antibody is associated with CSU disease duration [27]. In our study, only anti-TPO, not anti-Tg, predicted longer disease duration. Anti-TPO is indeed more sensitive and specific than anti-Tg for TA [37]. Moreover, anti-TPO also has superiority over anti-Tg for detecting AITDs such as Graves' disease and Hashimoto thyroiditis [38]. In addition, autoantibodies against the complement controller domain of TPO can activate complement through the classical pathway and raise C4a levels at baseline normalized in remission after treatment with levothyroxine. However, the potential role of anti-TPO in the pathogenesis of CSU remains to be determined [39].

The current study reinforced previous findings that ASST and/or APST could serve as a predictor for CSU severity including higher wheal number and more frequent attacks [26, 40, 41]. Interestingly, not only is the skin testing associated with CSU activity, but the wheal diameter could predict disease severity as well. Although, autologous skin testing did not show benefit in predicting disease duration, our results proved that APST can be used as a potential marker to predict difficult-to-treat cases requiring antihistamines exceeding standard dosing. Our results are in line with that of Staubach et al. proving that ASST positive patients were likely to use more antihistaminic medication than ASST negative patients [42]. Viswanathan et al. also demonstrated that commercially available basophil histamine release assay had strong association to refractoriness of antihistamines [20]. There is controversy regarding the advantage of APST and ASST over the other. Few studies have shown that APST gave higher percentage of positive result than ASST as coagulation factors in plasma might have a pivotal role in pathogenesis and severity of CSU [29, 43], whereas others have shown indifferences between them [44, 45]. Based on our finding, we believe that APST could be a parameter to predict therapeutic response in CSU; however, more research is needed to validate this finding

The limitation of this study includes its retrospective nature. Subjective evaluation of medical record was performed rather than using a validated instrument for disease severity such as the urticaria activity score [46]. Autoimmune CSU was confirmed by positive ASST and APST results, while BAT and histamine release test was not applied. Moreover, this study lacked the standard protocol for management. Step-wise algorithm for the treatment of CSU was not given to all patients; e.g., some patients were utilizing 2x daily antihistamine or more than one type of antihistamine, rather than the current recommended 4x dosing [46]. Finally, all patients were collected from a single center at a tertiary institution which may represent a more severe subgroup of CSU due to referral bias. However, the large sample size and the unified protocol for evaluation for all parameters conduced in this study should give more information regarding TA and autoimmunity in CSU linked to the disease severity and prognosis in CSU. Nevertheless, future prospective work is warranted to confirm our results.

In conclusion, we demonstrate that anti-TPO can potentially indicate longer CSU disease duration while autoimmunity in CSU can predict disease severity and therapeutic response. We recommend evaluating thyroid antibodies in patients with CSU particularly in females, patients > 35 years of age. Moreover, in all CSU patients especially < 35 years old, autologous skin testing is highly suggested.

## Figures and Tables

**Table 1:** Demographic data, severity of CSU, laboratory values, treatment regimens, disease duration according to the presence of thyroid autoimmunity, and results of thyroid autoantibodies.

Characteristics	Total	TA (+)	TA (-)	p-value	Anti-TPO (+)	Anti-TPO (-)	p-value	Anti-Tg (+)	Anti-Tg (-)	p-value
		n = 72	n = 228		n = 53	n = 247		n = 55	n = 245	
Gender [n = 300], n (%)				0.003*∗*			0.010*∗*			0.024*∗*
Male	46 (15.3)	3 (4.2)	43 (18.9)		2 (3.8)	44 (17.8)		3 (5.5)	43 (17.6)	
Female	254 (84.7)	69 (95.8)	185 (81.1)		51 (96.2)	203 (82.2)		52 (94.5)	202 (82.4)	

Age at onset (years) [n = 296], mean (SD)	41.3 (14.9)			0.008*∗*			0.187			0.006*∗*
≤ 35, n (%)		18 (25.0)	95 (42.4)		16 (30.2)	97 (39.9)		12 (21.8)	101 (41.9)	
> 35, n (%)		54 (75.0)	129 (57.6)		37 (69.8)	146 (60.1)		43 (78.2)	140 (58.1)	

Duration of symptoms (months) [n=291], median (range)	4 (1.5, 360)	3 (1.5, 120)	4 (1.5, 360)	0.434	3.3 (1.5, 120)	4 (1.5, 360)	0.840	3 (1.5, 120)	4 (1.5, 360)	0.524

History of NSAID hypersensitivity [n=300], n (%)	18 (6.0)	6 (8.3)	12 (5.3)	0.392	6 (11.3)	12 (4.9)	0.103	6 (10.9)	12 (4.9)	0.112

History of atopy [n = 129), n (%)	80 (62.0)	20 (64.5)	60 (61.2)	0.742	15 (65.2)	65 (61.3)	0.727	14 (56.0)	66 (63.5)	0.490

History of systemic symptoms [n = 195], n (%)	61 (31.3)	16 (35.6)	45(30.0)	0.481	15 (41.7)	46 (28.9)	0.137	12 (34.3)	49 (30.6)	0.672
Angioedema	61 (31.3)	15 (33.3)	46 (30.7)	0.735	14 (38.9)	47 (29.6)	0.276	11 (31.4)	50 (31.3)	0.984
Anaphylaxis	4 (2.1)	1 (2.2)	3 (2.0)	1.000	1 (2.8)	3 (1.9)	0.561	1 (2.9)	3 (1.9)	0.550

Dermographism [n = 186], n (%)	57 (30.7)	15 (34.9)	42 (29.4)	0.492	11 (33.3)	46 (30.1)	0.712	11 (37.9)	46 (29.3)	0.354

Duration of daily attacks (hours) [n=97], median (range)	3 (0.5, 24)	3 (0.5, 12)	3 (0.5, 24)	0.434	3 (0.5, 12)	3 (0.5, 24)	0.690	2 (0.5, 12)	3 (0.5, 24)	0.220

Frequency of attacks [n=216], n (%)				0.721			0.363			0.606
≤4 days per week	5 (25.5)	15 (27.3)	40 (24.8)		13 (31.0)	42 (24.1)		12 (28.6)	43 (24.7)	
>4days per week	161 (74.5)	40 (72.7)	121 (75.2)		29 (69.0)	132 (75.9)		30 (71.4)	131 (75.3)	

Wheal size [n = 79], n (%)				0.331			0.166			0.720
≤ 1.25 cm	20 (25.3)	2 (13.3)	18 (28.1)		1 (7.7)	19 (28.8)		2 (18.2)	18 (26.5)	
> 1.25 cm	59 (74.7)	13 (86.7)	46 (71.9)		12 (92.3)	47 (71.2)		9 (81.8)	50 (73.5)	

Number of wheals [n = 69], n (%)				0.748			0.740			0.283
≤ 7 wheals	23 (33.3)	5 (38.5)	18 (32.1)		3 (27.3)	20 (34.5)		5 (50.0)	18 (30.5)	
> 7 wheals	46 (66.7)	8 (61.5)	38 (67.9)		8 (72.7)	38 (65.5)		5 (50.0)	41 (69.5)	

Severity of itch^*∗∗*^ [n = 78], n (%)				0.729			0.469			1.000
None to mild	16 (20.5)	4 (25.0)	12 (19.4)		4 (28.6)	12 (18.8)		2 (15.4)	14 (21.5)	
Moderate to severe	62 (79.5)	12 (75.0)	50 (80.6)		10 (71.4)	52 (81.2)		11 (84.6)	51 (78.5)	

Impairment of work [n = 76], n (%)	54 (71.1)	10 (66.7)	44 (72.1)	0.754	8 (61.5)	46 (73.0)	0.504	9 (81.8)	45 (69.2)	0.494

Disturbance of sleep [n = 76], n (%)	51 (67.1)	9 (60.0)	42 (68.9)	0.549	8 (61.5)	46 (68.3)	0.748	6 (60.0)	45 (68.2)	0.721

High ESR [n = 133], n (%)	65 (48.9)	23 (63.9)	42 (43.3)	0.035*∗*	19 (67.9)	46 (43.8)	0.024*∗*	15 (62.5)	50 (45.9)	0.140

ANA titer ≥ 1:320 (+) [n = 232], n (%)	32 (13.8)*∗∗∗*	13 (23.6)	19 (10.7)	0.015*∗*	12 (29.3)	20 (10.5)	0.002*∗*	10 (23.3)	22 (11.6)	0.046*∗*

CAU (+) [n = 125], n (%)	101 (80.8)	20 (74.1)	81 (82.7)	0.316	17 (81.0)	84 (81.0)	1.000	14 (73.7)	87 (82.1)	0.363
ASST (+) [n = 125], n (%)	91 (72.8)	18 (66.7)	73 (74.5)	0.419	15 (71.4)	76 (73.1)	0.877	12 (63.2)	79 (74.5)	0.305
APST (+) [n = 125], n (%)	88 (70.4)	18 (66.7)	70 (71.4)	0.631	15 (71.4)	73 (70.2)	0.910	13 (68.4)	75 (70.8)	0.837

Treatment regimen, [n = 295], n (%)				0.667			0.296			0.819
H1 antagonist(s), standard dose	127 (43.1)	29 (40.9)	98 (43.8)		19 (36.5)	108 (44.4)		24 (44.4)	103 (42.7)	
H1 antagonist(s), any exceeding standard dose	168 (57.0)	42 (59.2)	126 (56.3)		33 (63.5)	135 (55.6)		30 (55.6)	138 (57.3)	

Disease duration > 12 months [n= 131], n (%)	112 (85.5)	28 (93.3)	84 (83.2)	0.240	22 (100.0)	90 (82.6)	0.042*∗*	17 (89.5)	95 (84.8)	0.739

Disease duration > 18 months [n= 101], n (%)	81 (80.2)	23 (92.0)	58 (76.3)	0.088	18 (100)	63 (75.9)	0.020*∗*	13 (86.7)	68 (79.1)	0.729

*∗∗* The definition of severity of itch: none = no itch, mild = present but not annoying or troublesome, moderate = troublesome but does not interfere with normal daily activity or sleep, severe = sufficiently troublesome to interfere with normal daily activity or sleep.

*∗∗∗* Considering ANA positivity at high titer (≥1:320), homogenous pattern was the most commonly observed (53.1%). Of 32 patients with high titer ANA, one had the validation to the diagnosis of systemic lupus erythematosus (SLE) via the Systemic Lupus International Collaborating Clinics Criteria with lupus nephritis class IVa, whereas the other had biopsy-proven subacute cutaneous lupus erythematosus.

*Abbreviations Used in Table 1*. ANA; anti-nuclear antibody (high titer at ≥ 1:320), Anti-Tg; anti-thyroglobulin antibody (normal range = 0-115 IU/mL), Anti-TPO; anti-thyroid peroxidase antibody (normal range = 0-34 IU/mL), APST; autologous plasma skin test, ASST; autologous serum skin test, CAU; chronic autoimmune urticaria, CSU; chronic spontaneous urticaria, ESR; erythrocyte sedimentation rate (normal range = 0-20 mm/hr), NSAID; nonsteroidal anti-inflammatory drug, TA; thyroid autoimmunity.

**Table 2:** Demographic data, severity of CSU, laboratory values including thyroid autoantibody results, treatment regimens, disease duration according to the presence of autoimmune chronic urticaria, and results of autologous skin testing.

Characteristics	CAU (+)	CAU (-)	p-value	ASST (+)	ASST (-)	p-value	APST (+)	APST (-)	p-value
	n = 101	n = 24		n = 91	n = 34		n = 88	n = 37	
Gender [n = 300], n (%)			0.740			1.000			0.580
Male	13 (12.9)	4 (16.7)		13 (14.3)	4 (11.8)		11 (12.5)	6 (16.2)	
Female	88 (87.1)	20 (83.3)		78 (85.7)	30 (88.2)		77(87.5)	31 (83.8)	

Age at onset (years) [n = 296], mean (SD)			0.027*∗*			0.145			0.087
≤ 35, n (%)	63 (62.4)	9 (37.5)		56 (61.5)	16 (47.1)		55 (62.5)	17 (46.0)	
> 35, n (%)	38 (37.6)	15 (62.5)		35 (38.5)	18 (52.9)		33 (37.5)	20 (54.1)	

Duration of symptoms (months) [n=291], median (range)	6 (1.5, 360)	11 (1.5, 120)	0.265	6 (1.5, 360)	11 (1.5, 120)	0.225	5 (1.5, 144)	8 (1.5, 360)	0.185

History of NSAID hypersensitivity [n=300], n (%)	4 (4.0)	2 (8.3)	0.325	4 (4.4)	2 (5.88)	0.663	3 (3.4)	3 (8.11)	0.360

History of atopy [n =129), n (%)	34 (59.7)	7 (41.2)	0.179	33 (63.5)	8 (36.4)	0.032*∗*	30 (57.7)	11 (50.0)	0.543

History of systemic symptoms [n = 195], n (%)	29 (34.5)	3 (14.3)	0.072	26 (33.8)	6 (21.4)	0.225	26 (35.1)	6 (19.4)	0.109
Angioedema	30 (35.7)	3 (14.3)	0.058	27 (35.1)	6 (21.4)	0.183	27 (36.5)	6 (19.4)	0.085
Anaphylaxis	1 (1.2)	0 (0)	1.000	1 (1.3)	0 (0)	1.000	1 (1.4)	0 (0)	1.000

Dermographism [n = 186], n (%)	21 (30.4)	9 (42.9)	0.290	19 (31.2)	11 (38.0)	0.523	20 (33.3)	10 (33.3)	1.000

Duration of daily attacks (hours) [n=97], median (range)	4 (0.5, 24)	2.5 (1, 8)	0.276	5 (0.5, 24)	2 (0.5, 12)	0.117	4 (0.5, 24)	3 (1, 12)	0.457

Frequency of attacks [n=216], n (%)			0.086			0.011*∗*			0.006*∗*
≤ 4 days per week	16 (18.4)	8 (36.4)		12 (15.4)	12 (38.7)		11 (14.7)	13 (38.2)	
> 4days per week	71 (81.6)	14 (63.6)		66 (84.6)	19 (61.3)		64 (85.3)	21 (61.8)	

Wheal size [n = 79], n (%)			0.716			0.318			0.759
≤ 1.25 cm	12 (23.1)	2 (14.3)		12 (25.5)	2 (10.5)		10 (22.7)	4 (18.2)	
> 1.25 cm	40 (76.9)	12 (85.7)		35 (74.5)	17 (89.5)		34 (77.3)	18 (81.8)	

Number of wheals [n = 69], n (%)			0.051			0.232			0.085
≤ 7 wheals	12 (26.1)	8 (57.1)		12 (28.6)	8 (44.4)		10 (25.6)	10 (47.6)	
> 7 wheals	34 (73.9)	6 (42.9)		30 (71.4)	10 (55.6)		29 (74.4)	11 (52.4)	

Severity of itch^*∗∗*^ [n = 78], n (%)			1.000			1.000			0.520
None to mild	11 (21.6)	3 (23.1)		10 (21.3)	4 (23.5)		8 (18.6)	6 (28.6)	
Moderate to severe	40 (78.4)	10 (76.9)		37 (78.7)	13 (76.5)		35 (81.4)	15 (71.4)	

Impairment of work [n = 76], n (%)	34 (65.4)	12 (85.7)	0.197	29 (61.7)	17 (89.5)	0.026*∗*	29 (65.9)	17 (77.3)	0.344

Disturbance of sleep [n = 76], n (%)	37 (71.2)	9 (64.3)	0.745	35 (74.5)	11 (57.9)	0.185	32 (72.7)	14 (63.6)	0.449

High ESR [n = 133], n (%)	15 (36.6)	4 (36.4)	1.000	15 (41.7)	4 (25.0)	0.249	14 (35.9)	5 (38.5)	1.000

ANA titer ≥ 1:320 (+) [n = 232], n (%)	9 (11.0)	0 (0)	0.357	8 (10.7)	1 (4.0)	0.444	8 (11.6)	1 (3.2)	0.267

TA (+) [n = 72], n (%)	20 (19.8)	7 (29.2)	0.316	18 (19.8)	9 (26.5)	0.419	18 (20.5)	9 (24.3)	0.631
Anti-TPO (+) [n = 53], n (%)	17 (16.8)	4 (16.7)	1.000	15 (16.5)	6 (17.7)	0.877	15 (17.1)	6 (16.2)	0.910
Anti- Tg (+) [n = 55], n (%)	14 (13.9)	5 (20.8)	0.363	12 (13.2)	7 (20.6)	0.305	13 (14.8)	6 (16.2)	0.837

Treatment regimen, [n = 295], n (%)			0.069			0.873			0.017*∗*
H1 antagonist(s), standard dose	41 (41.8)	15 (62.5)		40 (45.5)	16 (47.1)		33 (38.8)	23 (62.2)	
H1 antagonist(s), any exceeding standard dose	57 (58.2)	9 (37.5)		48 (54.6)	18 (52.9)		52 (61.2)	14 (37.8)	

Disease duration > 12 months [n= 131], n (%)	37 (84.1)	12 (85.7)	1.000	34 (85.0)	15 (83.3)	1.000	33 (84.6)	16 (84.2)	1.000

Disease duration > 18 months [n= 101], n (%)	23 (76.7)	10 (83.30	1.000	20 (76.9)	13 (81.3)	1.000	21 (77.8)	12 (80.0)	1.000

*∗∗* The definition of severity of itch: none = no itch, mild = present but not annoying or troublesome, moderate = troublesome but does not interfere with normal daily activity or sleep, severe = sufficiently troublesome to interfere with normal daily activity or sleep.

*Abbreviations Used in Table 2*. ANA: antinuclear antibody, Anti-Tg: antithyroglobulin antibody (normal range = 0-115 IU/mL), Anti-TPO: antithyroid peroxidase antibody (normal range = 0-34 IU/mL), APST: autologous plasma skin test, ASST: autologous serum skin test, CAU: Chronic autoimmune urticaria, ESR: Erythrocyte sedimentation rate (normal range = 0-20 mm/hr), NSAID = nonsteroidal anti-inflammatory drug, and TA = Thyroid autoimmunity.

**Table 3:** Information on demographics, diagnosis of and treatment for AITDs, and thyroid autoantibody results of patients with AITDs.

No.	Gender	Age at CSU onset (years)	Thyroid onset relative to CU and duration between thyroid diseases and CSU (months)	Diagnosis	Treatment	Anti-Tg (+)	Anti-TPO (+)
1	Female	17	Before, 108	Grave's disease	Oral medication, unspecified	Yes	Yes

2	Female	47	Before, 24	Grave's disease	Propylthiouracil	Yes	No

3	Female	62	Before, NA	Grave's disease	S/P I-131, Levothyroxine	No	No

4	Female	55	Before, 60	Hashimoto's disease	Levothyroxine	No	Yes

5	Female	41	Before, 21	Grave's disease	Methimazole	Yes	Yes

6	Female	48	Before, NA	Subclinical hypothyroidism	NA	Yes	Yes

7	Female	48	Before, 84	Grave's disease	Methimazole	No	Yes

8	Female	32	After, 24	Primary hypothyroidism	Levothyroxine	Yes	Yes

9	Female	31	After, 4.5	Grave's disease	MMI	No	Yes

10	Female	53	After, 1	Subclinical hyperthyroidism	Methimazole	Yes	Yes

11	Female	24	After, 22	Grave's disease	Methimazole	Yes	Yes

12	Female	17	After, 25	Hashimoto's disease	Levothyroxine	Yes	Yes

13	Female	30	After, 24	Grave's disease	Propylthiouracil	Yes	Yes

14	Female	12	After, 60	Unclassified	NA	Yes	Yes

15	Female	62	After, 1.5	Hashimoto's disease	Observe	Yes	Yes

16	Female	41	After, 1.5	Grave's disease	Methimazole	Yes	No

17	Female	46	After, 26	Grave's disease	Methimazole	Yes	No

18	Female	54	After, 21	Hashimoto's disease	Levothyroxine	Yes	Yes

19	Female	59	After, 2	Subclinical hypothyroidism	Levothyroxine	Yes	Yes

*Abbreviations Used in Table 3. *AITD: autoimmune thyroid disease, CSU: chronic spontaneous urticarial, and NA: no available data.

## Data Availability

The data used to support the finding of this study are available from the corresponding author upon request.
